# Evaluation of drug-drug interaction between daclatasvir and methadone or buprenorphine/naloxone

**DOI:** 10.7448/IAS.17.4.19628

**Published:** 2014-11-02

**Authors:** Tushar Garimella, Reena Wang, Wen-Lin Luo, Philip Wastall, Hamza Kandoussi, Michael Demicco, Douglas Bruce, Carey Hwang, Richard Bertz, Marc Bifano

**Affiliations:** 1 Research & Development, Bristol-Myers Squibb, Princeton, NJ, USA; 2 Research & Development, Anaheim Clinical Trials, Anaheim, CA, USA; 3 Clinical Investigation, Yale University, New Haven, CT, USA

## Abstract

**Introduction:**

Daclatasvir (DCV) is a potent hepatitis C virus (HCV) NS5A replication complex inhibitor with pangenotypic (1–6) activity in vitro. Methadone (MET) and buprenorphine (BUP) are opioid medications used to treat opioid addiction; patients on HCV therapy may require MET or BUP treatment. The effect of DCV on the pharmacokinetics (PK) of MET or BUP/naloxone (NLX) was assessed in subjects on stable MET or BUP.

**Materials and Methods:**

An open-label, two-part study assessed the effect of steady-state oral administration of DCV on the PK of MET (Part 1, P1) or BUP/NAL (Part 2, P2). Safety/tolerability and pharmacodynamics (PD, opioid withdrawal scales/overdose assessment) were also assessed. Subjects (P1, N=14; P2, N=11) received daily single-dose oral MET (40–120mg) or BUP/NLX (8/2–24/6mg) based on their prescribed stable dose throughout, in addition to DCV (60mg QD) on Days 2–9. Serial PK sampling occurred predose and postdose till 24 hours on Day 1 (MET/BUP) and Day 10 (MET/BUP/DCV). Noncompartmental PK were derived. Geometric mean ratios (GMR) and 90% confidence intervals (90% CI) for MET/BUP/norBUP C_max_ and AUC_TAU_ were derived from linear mixed effects models.

**Results:**

Subjects were aged 19–39 years, mostly white (P1, 93%; P2, 100%) and male (P1, 71%; P2, 91%). All subjects completed the study. No clinically meaningful effect was demonstrated as the GMR and 90% CIs fell within the prespecified interval (P1, 0.7–1.4; P2, 0.5–2.0: see Table 1). DCV coadministration was well-tolerated: overall, six (43%) subjects had adverse events (AEs) (all mild and resolved without treatment). DCV had no clinically significant effect on the PD of MET or BUP/NLX.

**Conclusions:**

Steady-state administration of DCV 60mg QD had no clinically meaningful effect on the PK of MET or BUP/NLX and was generally well-tolerated, suggesting that no dose adjustments will be required.

**Table 1:** Pharmacokinetic parameters and statistical comparisons

		With DCV	W/O DCV	GMR
		
		Adj. Geo. Mean	Adj. Geo. Mean	(90% CI)
R-MET^a^	C_max_, ng/mL	103.6	96.6	1.07 (0.97, 1.18)
	AUC_TAU_, ng•h/mL	1699.6	1569.8	1.08 (0.94, 1.24)
BUP	C_max_, ng/mL	3.3	2.5	1.30 (1.03, 1.64)
	AUC_TAU_, ng•h/mL	25.1	19.2	1.31 (1.15, 1.48)
NORBUP	C_max_, ng/mL	3.1	1.8	1.65 (1.38, 1.99)
	AUC_TAU_, ng•h/mL	42.6	26.4	1.62 (1.33, 1.96)

